# Human molecular genetics sheds light on the physiological significance of ribonuclease inhibitor (RNH1)

**DOI:** 10.1038/s41431-023-01362-4

**Published:** 2023-04-21

**Authors:** Mayuresh Anant Sarangdhar, Nicola Andina, Ramanjaneyulu Allam

**Affiliations:** 1 Department of Hematology and Central Hematology Laboratory, Inselspital, Bern University Hospital, University of Bern, Bern, Switzerland; 2 Department for BioMedical Research, University of Bern, Bern, Switzerland

**Keywords:** Disease genetics, Genetics research

Ribonuclease inhibitor (RI or RNH1), also known as angiogenin inhibitor 1 (OMIM 173320), is a 50-kDa protein that is expressed throughout the human tissues [1], with particularly high levels found in myeloid cells [2]. While it is mainly localized in the cytosol, it can also be found in the nucleus and mitochondria [3]. It is composed entirely of leucine-rich repeats (LRRs). In fact, RNH1 is the first cytosolic protein identified to possess LRRs [4, 5]. Phylogenetically, RNH1 evolved via exon duplication and is conserved among mammals [1]. RNH1 was discovered around the 1960s in the supernatant fraction prepared from rat liver homogenates, with ability to bind and inhibit pancreatic-type RNases (ptRNases) [6, 7] such as RNase A, RNase 1, RNase 2, RNase 4 and angiogenin (ANG, also known as RNase 5). Since then, RNH1 became a popular protein amongst biochemists for its ability to inhibit ribonucleases. RNH1 is frequently employed in in vitro biochemical reactions to safeguard RNA from degradation by unintended RNases. In addition, several other biological functions of RNH1 have been reported, such as involvement in cancer growth and metastasis [8], tiRNA degradation [9, 10], microRNA (miR-21) processing [11], differentiation and myelination of oligodendrocytes [12] and inhibitor of oxidative damage [1, 13]. Recent studies from *Rnh1* knockout mice revealed that RNH1 is important for embryonic development [14], mRNA translation [15], hematopoiesis [14] and inflammation [2] and raised questions about the significance of RNase inhibitor function of RNH1 in vivo. Despite its routine application and extensive research over the last 50 years aimed at understanding its structure and functions, the exact significance of RNH1 in human physiology remains unknown. Furthermore, the name “Ribonuclease inhibitor” is too specific for its broad range of functions, downplaying other important roles of RNH1.

**Fig. 1:**
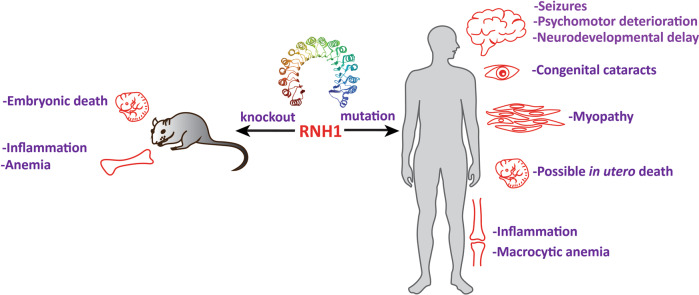
Human and mouse molecular genetics have uncovered important physiological functions of Ribonuclease inhibitor 1 (RNH1). Constitutive *Rnh1* knockout in mice is embryonically lethal, shows erythroid differentiation defects and anemia phenotype. *Rnh1* deletion in myeloid cells increases inflammation by innate immune activators. Loss-of-function mutations in the human *RNH1* gene affects brain, muscles, eyes and cause infection-induced psychomotor deterioration, seizures, and macrocytic anemia. RNH1 structure is taken from PDB.

Hedberg-Oldfors et al. describe for the first time the human phenotype resulting from a loss-of-function mutation in *RNH1* [16]. The mutation creates a splice-site variant *(c.615-2A* > *C)* in the *RNH1* gene, which leads to aberrant splicing of exons with skipping of exon 7 and loss of full-length RNH1 protein in humans. This mutation was detected in a family of seven children born to healthy first-cousin parents of Somali origin. In this family, authors report two cases of neurodevelopmental delay, myopathy, congenital cataracts and infection-induced psychomotor deterioration, seizures, and macrocytic anemia (Fig. 1). The parents were healthy, heterozygous carriers of the mutation. Of their eight offspring, two were homozygous (a boy and a girl) and affected, three were heterozygous and apparently healthy, and one died in utero at week 20 of gestation, and was not tested for the *RNH1* mutation. The affected boy died at the age of 8 months while the girl survived with global developmental delay.

Taken together, *RNH1* deficiency in humans seems to cause problems only in a homozygous state, whereas heterozygous carriers are typically healthy. *RNH1* deficiency has a rather dramatic phenotype, manifesting shortly after birth or even already in utero, such as bilateral cataracts observed in one sibling. The affected tissues include neurons, muscles, eye lens, erythropoiesis, and the immune system. Based on the presented data, erythropoiesis at steady state is probably sufficient, but it deteriorates during infection, which hints to a defective stress-erythropoiesis [16].

It’s worth noting that a previous study in mice provided some similar in vivo phenotypic evidences [14]. First, only homozygous *Rnh1*-deficient mice were affected, while heterozygous carriers remained healthy. Second, all affected mice died in utero, and therefore the onset was again early in their development. Third, erythropoiesis was reduced but still present in mouse embryos (Fig. 1). However, the potential defects in neurons, muscles and eye lenses could not be examined in these E8.5 to E10 mouse embryos and may need further attention. In addition, Bombaci et al. reported a haematopoietic-specific conditional *Rnh1*^*−/−*^ mouse model that bypassed the vulnerable embryonic phase [2]. They are viable and do not show gross phenotypic defects. Interestingly, upon challenge with small doses of lipopolysaccharide (LPS), all conditional *Rnh1*-deficient mice died, whereas their wild-type littermates survived. This suggests that these mice struggle to cope with even mild immunological challenges. These findings are similar to those observed in the two reported human siblings who had up to five hospitalizations upon infection during their first year of life.

There were few interindividual as well as interspecies (between mouse and human) differences in *RNH1* mutant phenotypes. The interindividual difference includes severity of disease where one of the affected siblings died at the age of 8 months while the other survived. This difference in severity was also observed between species: Constitutive *Rnh1* knockout was completely lethal in utero for mice but two *RNH1*-deficient patients survived embryonic development. This variability in severity could potentially be attributed to a compensatory mechanism involving a truncated version of the RNH1 protein although such a protein was not detected by antibodies and in cell-type used by the authors. Moreover, presence of other disease-causing genetic mutations cannot be entirely ruled out that may have contributed to the observed differences. Despite these differences in severity, the shared phenotype described in patients is consistent with the one observed in *Rnh1* knockout mice, strongly arguing for a causative role of *RNH1* deficiency in the pathogenesis.

To explain the mechanism behind RNH1-mediated pathophysiology, the authors focused on the “Ribonuclease inhibitor” function of RNH1 [16]. Pancreatic-type RNases can enter cells and degrade RNAs leading to cell death or tissue damage [1]. Therefore, loss of RNH1 could increase RNase-mediated tissue damage. Supporting this Hedberg-Oldfors et al. showed increased cell death in *RNH1* mutated patient-derived fibroblasts treated with increased concentration of RNase A compared to healthy control [16]. However, these results should be interpreted with caution because: (1) RNase A is a bovine counterpart for human RNase 1 and has ~5 fold more activity than human RNase 1 [17]. (2) No cell death was observed in patient-derived fibroblasts when RNase A was added at lower concentrations or not added at all. At higher concentrations (about 200–1000 times higher than ~0.5 ug/ml of RNase 1 usually present in blood and other body fluids [17, 18]) of RNase A, only ~15–20% of cell death was reported. This suggests that, under normal conditions, cells with a deficiency in RNH1 can still protect their RNAs and survive. (3) There is no data that RNase activity is increased in serum and cerebrospinal fluid of these patients. (4) RNase 1 is widely expressed in several tissues [17, 19] but *RNH1* mutated patients did not show multi-organ damage. Rather, they have specific phenotypes such as neurodevelopmental delay, myopathy, congenital cataracts and anemia. (5) RNA sequencing analysis of patient-derived fibroblasts, yolk-sac-derived cells from *Rnh1* knockout mice, as well as *Rnh1* knockout K562 cells, did not show large changes in the transcriptome compared to their respective controls [14, 16] (6) *RNase 1* knockout mice do not show an abnormal phenotype and have high extracellular RNA levels (eRNA) [20]. eRNA activates innate immune response [21], however these mice did not show any inflammatory phenotype despite increased eRNA levels. Although an increase in RNase activity in *RNH1* mutant patients could potentially decrease eRNA levels and reduce inflammation, these patients, in fact, experience more inflammatory episodes. Collectively, it is less likely that RNase-mediated toxic effects contribute to the disease phenotype in these patients. Rather, cell intrinsic functions of RNH1 might play an important role.

Further studies are still needed to check the extent to which RNases and mutated RNH1 contribute to the pathogenesis of the disease. For this, thorough characterization of the immune function and long-term follow-up of patients is needed. Additionally, generating *RNH1* and *RNases* double knockout mice would be helpful to address these points.

Though there are several questions needed to address, this study by Hedberg-Oldfors et al. reveals an important role for RNH1 in human pathophysiology and motivates further studies on RNH1 [16]. Identifying more *RNH1* mutations in humans and in combination with functional and genomic studies will unravel the precise role of RNH1 in human physiology.
